# Efficiency evaluation and nonlinear multi-objective optimization of forestry industry transformation in the Heilongjiang state-owned forest region

**DOI:** 10.1038/s41598-023-47953-5

**Published:** 2023-12-01

**Authors:** Shuo Diao, Yude Geng

**Affiliations:** College of Economics and Management, Northeast Forestry University, Harbin, 150040 China

**Keywords:** Biogeochemistry, Ecology, Environmental sciences, Environmental social sciences, Mathematics and computing, Nanoscience and technology, Physics

## Abstract

With the deepening of the concept of sustainable development of the whole society, protecting forest resources has become a crucial task of the current society. The present forestry industrial structure of Heilongjiang state-owned forest areas has undergone significant changes, and the transformation of the forestry industry has become increasingly prominent. How to deepen the forestry industry transformation and improve its efficiency has become an important research direction in forest areas. This work first analyzes the data envelopment method, and further designs the evaluation method of forestry transformation efficiency in forest areas. Then, the evaluation index system of forestry industry transformation efficiency in Heilongjiang state-owned forest areas is built. The relevant nonlinear multi-objective optimization (MOO) constraints are designed. Relevant data are quoted to evaluate the efficiency of the forestry industry transformation in the Heilongjiang state-owned forest areas. The results show that: (1) During 2015–2021, the average value of the scale, technical, and comprehensive production efficiencies of Heilongjiang state-owned forest areas were 0.765, 0.53, and 0.399, all of which were less than 1. And they were in a relatively ineffective state. (2) The overall industrial transformation of state-owned forest areas was optimistic. The technical efficiency decreased slightly in 2017, while the pure technical efficiency was greater than 1 in 2016 and 2018. The efficiency value increased to the peak by the end of 2021. (3) In the transformation of the forestry industry in state-owned forest areas, the influence of the industrial economy and resource protection subsystems was the first and backward, and the contribution of the social development subsystem was in the middle. (4) In the MOO problem, the forest area should be planned according to the proportion of public welfare, multi-functional, and commercial forests: 35.2%, 38.8%, and 26%, respectively. This work provides an essential reference for protecting forest resources and contributes to the transformation and development of the social forestry industry.

## Introduction

With continuous social progress, the concept of green sustainable development (SD) has become the main development direction of the current society. Hence, protecting forest resources has also become vital to promote society’s overall development.

Relying on unique natural resources, the Heilongjiang state-owned forest areas contributed a lot to the early economic construction of China. In the 1950s, the state-owned forest areas of Heilongjiang Province established the initial forest management mode under the ideological system, with the production of wood resources as the core^1^. Although the structure is unitary, Heilongjiang Province ranks first in the national economic output by resource advantages during the first 5-year plan period. However, with the development of the requirements of the times, the problem of forestry industry transformation rooted in the resource-based economy development mode and the “integration of government and enterprises” system has not been completely solved. Moreover, the massive impact of the comprehensive logging cessation policy on forest areas makes the current forestry industry transformation path unable to meet the effective implementation of the forestry industry transformation process^2^. Besides, the Heilongjiang state-owned forest areas still need to achieve the transformation of the forestry industry and the optimization of the forestry industrial structure overnight. The transformation path of the new forestry industry needs to be optimized and adjusted based on the old path^3^. The appropriate path can bring benign promotion for the forestry industry transformation in state-owned forest areas of Heilongjiang Province and even SD. Therefore, it is particularly essential and urgent to research and determine the transformation path of the new forestry industry in state-owned forest areas of Heilongjiang Province. It is also a significant development direction to provide technical support for protecting the overall forest resources of society through research^4^.

Currently, it is quite urgent for the whole society to study the forestry transformation, which can improve the development effect of forestry, and provide support for the broad application of the concept of green and SD in society. Forestry transformation belongs to the transformation at the micro level, which refers to the cyclical process of the forestry industry. In terms of time, the transformation of the forestry industry is the continuous periodic change of various production factors between industries and enterprises^5^. In space, it is the constant change, transfer, and aggregation of various production factors, forestry departments, and forestry enterprises in space and region^6^. The data envelopment analysis (DEA) method is more commonly used in forestry transformation efficiency analysis. This method can measure and evaluate the efficiency of the input scale and technical effectiveness. It means that after investing a certain amount of capital, labor, and other elements in the same type of enterprise. Furthermore, it also can evaluate the relative effectiveness of their output benefits^7^. DEA is adopted to calculate the efficiency of forestry industry transformation efficiency to further measure the standard and process of forestry industry transformation. Meanwhile, with the progress of the overall social market economy and optimization of the forestry industry management system in state-owned forest areas, the oil field development has shifted from production-centered to benefit-centered. The level of economic benefits has become one of the vital bases for forest development decision-making. Benefits planning is the optimal planning to achieve multiple goals^8^.

Based on the above analysis, the research goal will be listed to promote society's overall green and SD. The first is how to deepen the transformation of the forestry industry and choose a reasonable path to transformation to make the SD strategy become a generally accepted way in state-owned forest areas.

Then, the current situation of forestry industry transformation in Heilongjiang state-owned forest areas is further explored. Choosing and optimizing the transformation path of the forestry industry is conducive to objectively judging the transformation efficiency and influencing factors at the current stage. It can provide a basis for determining the transformation path of the forestry industry in state-owned forest areas of Heilongjiang Province in the period of reform and transformation. Moreover, it can promote the forestry industry's stable and accelerated transformation process. Meanwhile, according to the actual situation of forestry in the forest areas, the forestry industry in the state-owned forest areas is divided into different parts. A nonlinear multi-objective optimization (MOO) model of profit, output, cost, and investment is established to realize the optimal decision-making of benefits planning. This work can provide reference suggestions for the transformation of the forestry industry of the whole society, and also contribute to the improvement of the concept of green SD of the whole society.

## Literature review

Many researchers studied the current methods of forest resources to improve the effect and ability of SD of society. Among them, Zhu and Song (2021) discussed the common strategy of developing artificial forests adopted by all countries in the world to solve the problem of decreasing natural forests. They regarded vigorously developing artificial forests as the fundamental measure to solve the demand for environment and timber in the twenty-first century. The revision of forestry policies and the reform of forestry management institutions were significant measures being taken or planned by many developing countries and countries with economic system transition^9^. Wolf et al.^10^ proposed that SD was a scientific development concept that focused on the sustainable use of the ecological environment and resources while developing the economy. This concept ran through all fields of social development. The current situation and characteristics of social forest resources require SD, continuous improvement of the protection and management of forest resources, and ultimately the sustainable use of resources for the benefit of future generations^10^. Gu et al.^11^ pointed out that natural forest protection, conversion of farmland to forests, and other forestry policies have contributed to mitigating climate warming. However, the carbon sink of these forest resources cannot be traded due to the restrictions of afforestation and reafforestation project rules. It was proposed to establish a voluntary carbon emissions trading market, and promote the entry of forest resources into the trading framework in a broader sense through the design of the trading mechanism^11^. The purpose was to integrate forest restoration, protection, and climate change issues and ensure social economy security and the full play of the functions of the forest eco-economic system. Onufrey and Bergek^12^ deemed that with rapid social progress, the impact of human beings on the environment was increasing. Excessive deforestation led to global warming, and humankind tasted the consequences. Forestry can balance the forest ecosystem’s development and ensure the harmonious coexistence of humans and nature. They believed the forestry industry was a special complex industrial group with a wide range of products and a long industrial chain. Based on this, the five driving factors of the transformation and upgrading of the forestry industry were analyzed, including the natural conditions, the deep motivation, the internal reasons, the cultural motivation, and the external impetus. They proposed strengthening the cooperation among forestry enterprises and building a service platform for the forestry industry. Scientific and technological innovation in the forestry industry should be accelerated, giving play to the government's public service function and promoting the transformation and upgrading of the forestry industry^12^. Wang et al.^13^ pointed out that industrial transformation and upgrading were the basic means to transform the mode of economic growth, improve economic development efficiency, and achieve rapid and sound economic development. Only through industrial transformation can people completely solve the problems faced by resource-based industries. The industrial transformation was the only way for the state-owned forest industry to develop to a higher level. The healthy and stable development of the forestry industry was a crucial guarantee for promoting forestry production efficiency, the living conditions of forest residents, the income of forestry workers, and the coordinated development of the economy, society, resources, and environment^13^. To sum up, the current forest resources had been destroyed, so optimizing forest resource protection measures was one of the crucial tasks in social development. The forestry industry transformation was a measure that can optimize the forestry industry and ensure the sustainable use of forest resources. However, the current research was not mature, so many forestry industries had not been completely transformed, facing huge social challenges.

In summary, the common contribution of these documents was to emphasize the importance of forest resource management and forestry industry transformation for social SD. They proposed strategies and ideas to address the decline of forest resources, promote SD, mitigate climate warming, and improve economic efficiency. Topics covered include developing plantation forests, improving conservation and management measures, establishing carbon emission trading markets, enhancing cooperation between enterprises and technological innovation, and promoting industrial transformation and upgrading. However, these documents also had some shortcomings. First, they lacked specific implementation details and case studies, and needed to fully explore the implementation effects and challenges of the proposed strategies and recommendations. Second, these opinions and conclusions here sometimes need more in-depth theoretical or empirical research support and a comprehensive discussion of relevant issues. In addition, some literature only provided a macro-level view and required a detailed discussion of specific policies and practices. Although these documents provided some crucial perspectives and strategies for forest resource management and forestry industry transformation, more research was needed to explore these issues in depth. Specific implementation plans and case studies should be provided to promote forest resource conservation and sustainable use and achieve social development goals. Based on the above content, to promote the transformation of the forestry industry and effectively protect forest resources, this work was committed to studying the transformation and development status of the forestry industry and providing a reference for its future development.

## Methods

### Industrial transformation status of Heilongjiang state-owned forest areas

Heilongjiang Province is China's largest province of forestry resources, with vast forest resources and rich forestry potential. However, the state-owned forest areas in Heilongjiang are faced with challenges and problems due to over-reliance on the traditional resource-based industry model and forestry management mode for a long time.

1. Transformation status.

The structure of the forestry industry in the Heilongjiang state-owned forest area is relatively unitary, mainly focusing on timber harvesting and processing, and lacking high-value-added forestry products and services. This single industrial structure makes the forestry industry more vulnerable to market competition.

2. Waste of resources and environmental pressures.

In the past development process, some state-owned forest areas have had problems with the waste of resources and environmental damage. For example, excessive deforestation and indiscriminate logging have led to the reduction of forest resources and the degradation of ecosystems, while increasing the pressure on the ecological environment.

3. The management system is not perfect.

In the state-owned forest area, the traditional forestry management mode has some drawbacks, such as the inflexible decision-making mechanism, the relatively backward management means, the need for more scientific and technological innovation and technology introduction. These problems limit the development and efficiency potential of the forestry industry.

(2) Problems to be solved in the transformation.

1. Industrial diversification and upgrading.

How to promote the diversification of the forestry industry structure, develop and produce forestry products and services with higher added value, and improve the competitiveness of state-owned forest areas and SD ability.

2. Conservation and sustainable use of resources.

How to strengthen the protection and management of forestry resources, realize the sustainable use of resources, reduce excessive felling and deforestation, restore and improve the ecological environment, and ensure the sustainable supply of resources.

3. Innovative technology and management mode.

How to introduce advanced science and technology and management mode, improve the forestry operation efficiency and management level of state-owned forest areas, strengthen technological innovation and technology transfer, and improve production efficiency and product quality.

4. Promotion of enterprise cooperation and coordinated development of industrial chain.

How to strengthen the cooperation between enterprises inside and outside the state-owned forest areas, construct an excellent industrial cooperation network and supply chain, realize resource sharing, mutual benefit, and win–win. At the same time, how to promote the coordinated development of the upstream and downstream links of the industry chain, and improve the comprehensive competitiveness and value-added ability of the whole forestry industry.

5. Policy support and institutional innovation.

How to formulate and implement policies and measures conducive to the transformation of the forestry industry, covering fiscal and tax support, land policies, financial support, and other aspects, to provide policy support and guarantee for the industrial transformation of state-owned forest areas. At the same time, it is also necessary to innovate the forestry management mechanism, make the decision-making more scientific and flexible, strengthen the supervision and evaluation mechanism, and ensure the effective implementation and implementation of the transformation policy.

6. Personnel training and technological innovation.

How to train and introduce the talents and professional and technical personnel needed to transform the forestry industry and improve the state-owned forest area's innovation ability and technical level. Meanwhile, it needs to strengthen the cooperation between scientific research institutions and enterprises, promote technological innovation and the transformation and application of research and development (R&D) results, and improve the technological content and competitiveness of the forestry industry.

7. Participation in social governance and public participation.

How to strengthen communication and cooperation between state-owned forest areas and local communities, the public, and stakeholders, establish a good social co-governance mechanism, and achieve social consensus and SD of forestry industry transformation.

### DEA methods

DEA belongs to operations research. It is a way to explore the boundary of economic production. It is usually adopted to measure the production efficiency in some departments responsible for making decisions^14^. It is to use the linear programming method on the basis of multiple input and output indicators to assess the relative effectiveness of the same type of units with comparability, which is a quantitative analysis method. The DEA method and its model are widely applied in various industries and departments^15^. The DEA method includes the CCR model with the constant return to scale (CCR is the abbreviation of three operational research scientists, namely A. Charnes, W. W. Cooper, and E. Rhodes) and the BCC model with the variable return to scale. CCR and BCC are to calculate comprehensive and pure technical efficiency (PTE)^16^.

The CCR model has clear ideas, a simple model form, and a relatively perfect theory. If there are *n* Decision Making Unit (DMUs), and each DMU has m inputs and s outputs, the output vector of decision unit *j* is:

1 $$ X_{j} = \left( x \right._{1j} ,.x_{2j} , \ldots ,\left. {x_{mj} } \right)^{T} $$

${x}_{ij}$ refers to the *i*-th input of the *j*-th DMU^17^, and the output vector of the decision-making unit is:

2 $$ Y_{j} = \left( y \right._{1j} ,.y_{2j} , \ldots ,\left. {y_{sj} } \right)^{T} $$

${y}_{sj}$ is the *s*-th output of the *j*-th DMU^18^. The input, as well as output weight vectors of the decision-making unit reads:

3 $$ {\text{u}} = \left( {u_{1} ,u_{2} , \ldots ,u_{m} } \right)^{T} $$

4 $$ {\text{v}} = \left( {v_{1} ,v_{2} , \ldots ,v_{s} } \right)^{T} $$

$v_{i}$. stands for the weight of the i-th input. $u_{m}$ refers to the wght of the *m*-th output^19^.

The mathematical model for evaluating the efficiency of the decision-making unit is as follows:

5 $$ {\text{max}}\frac{{u^{T} Y_{j0} }}{{v^{T} X_{j0} }} $$

6 $$ {\text{s}}.{\text{t}}.\left\{ {\begin{array}{*{20}c} {\frac{{u^{T} Y_{j} }}{{v^{T} X_{j} }} \le 1, j = 1,2, \ldots ,n} \\ {u \ge 0,v \ge 0,u \ne 0,v \ne 0} \\ \end{array} } \right\} $$

### Evaluation method of transformation efficiency of the forestry industry in forest areas

The DEA method is adopted for measuring the forestry transformation efficiency of Heilongjiang state-owned forest areas. This method calculates and evaluates the efficiency of the input scale and the technical effectiveness of its decision-making unit. It aims to assess the relative effectiveness of the output of the same type of enterprise after inputting specific capital, labor, and other elements^20^. The DEA model method calculates the input–output efficiency, that is, the production efficiency. Forestry input–output efficiency can adequately reflect the effect of the forestry industry transformation. Thus, the forestry input–output efficiency calculated by the DEA model can be replaced by industry transformation efficiency. The efficiency of forestry industry transformation is the basis for measuring the standard and process of forestry industry transformation. It is employed to represent the transformation efficiency of the forestry industry^21^.

The BCC model with the variable return to scale is selected to calculate the transformation efficiency of the forestry industry. To obtain the efficiency value of the decision-making unit, it is necessary to calculate *n* linear programming problems to calculate the input (${x}_{j}$), weight (${v}_{j}$), output (${Y}_{i}$), and its weight (${U}_{i}$). Assuming that *m* inputs and *S* outputs exist, the fractional programming model is transformed into a linear programming model^22^.

According to BCC input–output indicators, the comprehensive, technical, and scale efficiency of forestry industry transformation in state-owned forest areas of Heilongjiang Province from 2015 to 2021 are obtained. The efficiency value is distributed in the interval (0,1]. When the efficiency value of DMU is 1, it is at the forefront of efficient production. If it is less than 1, production is inefficient, and its relative ineffectiveness is further judged according to the difference with 1. Comprehensive efficiency (t) is a comprehensive evaluation of multiple indicators, such as the rational allocation of forestry resources and the input–output efficiency of forestry production factors in forestry industry transformation in the Heilongjiang state-owned forest areas^23^. Technical efficiency (p) is the comprehensive efficiency of forestry enterprises affected by forestry technology level, innovation ability, and other factors. Scale efficiency (s) is the comprehensive efficiency of forestry enterprises affected by the scale of forestry management. PTE and scale efficiency are the specific decomposition of the comprehensive efficiency of the forestry industry transformation. Besides, they are the effective evaluation of the transformation efficiency of the forestry industry in the Heilongjiang state-owned forest areas from different angles^24^.

Generally, the DEA model has two forms. The input-oriented model is to measure the efficiency with the least input factors when the output variables are fixed. Output-oriented mode is to measure efficiency by maximizing output variables without changing input factors^25^. The overall economic progress of the Heilongjiang state-owned forest areas is relatively backward, directly restricting the transformation's financial support. The serious population loss leads to a lack of labor, and the accelerated aging process increases the social burden. These factors limit the input of factors to a great extent. Therefore, this work chooses the DEA method of input-oriented mode. It is to reduce factor input under the premise of constant output to improve transformation efficiency.

### Establishment of evaluation system of forestry industry transformation efficiency in state-owned forest areas of Heilongjiang

According to the actual situation of the transformation of key state-owned forest areas in Heilongjiang, from the perspective of the overall development of the industrial economy, social development, and resource protection, the transformation efficiency evaluation index system of the key state-owned forest areas is standardized in accordance with the principles of systematization, typicality, dynamics, simplicity, scientificity, and accessibility of the index system (Table 1):

**Table 1:** Evaluation index system of transformation efficiency of key state-owned forest areas in Heilongjiang Province.

Primary indicators	Secondary indicators
Input indicators	R&D and training expenditure (X1)
	Ecological protection and environmental investment (X2)
	Investment in wood and forest facilities (X3)
	Tourism, leisure, and understory economic expenditures (X4)
	Rural economic transformation support investment (X5)
Output indicators	Total forestry output value (Y1)
	Output value of wood and forest products (Y2)
	Output value of tourism and leisure services (Y3)
	Output value of green energy (Y4)
	Ecological protection benefits (Y5)
	Output value of the tertiary industry (Y6)
	The growth of farmers' income (Y7)
	Enhancement of employment opportunity (Y8)
	Forest resource reserve (Y9)
	The achievements of technological innovation and investment return (Y10)

Table 1 exhibits that the evaluation index system of transformation efficiency of key state-owned forest areas in Heilongjiang Province is mainly divided into the following two aspects:

(1) Input indicators: The input indicators’ selection is based on the key needs of the forestry industry transformation in the state-owned forest areas, including specific resource inputs, such as R&D and training expenditure, to enhance the capacity of technology and human resources to promote innovation and technology upgrading. Ecological protection and environmental investment are aimed at maintaining ecological balance and ensuring sustainable utilization of resources. Investment in wood and forest facilities is used to upgrade and maintain forestry facilities to improve production capacity and quality. Tourism, leisure, and understory economic expenditures are aimed at promoting diversified economic development in forest areas, creating employment, and increasing income levels. Rural economic transformation support investment provides financial support for rural areas, helps farmers achieve employment opportunities and increase income, and promotes rural social stability and development^26,27^.

(2) Output indicators: The output indicators’ selection considers the key objectives and performance evaluation needs of forestry industry transformation in state-owned forest areas, involving measuring the total output value of forestry to reflect the contribution of the entire forestry industry. The output value of wood and forest products highlights the economic importance of forest products. The output value of tourism and leisure services emphasizes the tourism potential of forest areas and the contribution of eco-tourism. The output value of green energy, which emphasizes the production and utilization of sustainable energy, is related to environmental friendliness. Ecological conservation benefits measure ecosystem health and resource conservation outcomes. The output value of tertiary industry reflects the development of service industry and the promotion of diversified economy. The growth of farmers' income focuses on the well-being of rural residents and economic improvement. Enhancement of employment opportunity emphasizes the importance of job creation, especially in rural areas. Forest resource reserves, taking into account future resource sustainability and long-term conservation. The achievements of technological innovation and investment return are evaluated to ensure the SD of the industry. These indicators comprehensively reflect the multi-dimensional performance and social and economic benefits of the forestry industry transformation in state-owned forest areas, and help to comprehensively evaluate the legitimacy and sustainability of the industry^28,29^.

After the selection of evaluation indicators, the index weight is further calculated. When constructing the evaluation model, to avoid the problem that the subjective idea in the subjective weighting method may lead to the instability of the weight, the entropy weight method is adopted for objective weighting. Technique for Order Preference by Similarity to an Ideal Solution (TOPSIS) is a method to approximate the ideal solution and rank it by the closeness between each evaluation scheme and the ideal goal.

The steps of weighting the evaluation indicators follows: (1) it is the dimensionless processing of the original data; (2) it is to calculate the information entropy of the data, as illustrated in Eq. (7):

7 $$ e_{i} = - \left( {\mathop \sum \limits_{i = 1}^{m} P_{i} lnP_{i} } \right)/ln\left( n \right) $$

(3) The index weight is further calculated according to the information entropy, as shown in Eq. (8):

8 $$ V_{ij} = X^{\prime}_{ij} \times w_{i} $$

(4) The calculated weight value is extracted and processed to form positive as well as negative ideal solution vectors. (5) It calculates the weight value and the distance between the positive and negative ideal solutions. (6) The forestry industry's transformation efficiency evaluation index T is calculated in state-owned forest areas. Meanwhile, the evaluation index is divided into five levels: [0,4], which means the transformation efficiency is extremely poor; (0.4,0.6] means the transformation efficiency is poor; (0.6,0.7] means the transformation efficiency is qualified; (0.7,0.8] means the transformation efficiency is intermediate; (0.8,1] means the transformation efficiency is excellent.

### The nonlinear MOO problem

In the previous section, this work evaluated the transformation efficiency of industries in Heilongjiang state-owned forest areas to some extent, analyzed the current input–output relationship, and identified the existing problems and room for improvement. Then, this section uses the nonlinear MOO method to find an optimal solution set by setting appropriate objective functions and constraints to maximize output indicators under given constraints.

For the forestry industry in Heilongjiang state-owned forest areas, the decision-making of nonlinear multi-objective management planning is carried out, that is, to adjust the structure of the forestry industry in state-owned forest areas. First, the optimization model of forest ecosystem structure is adopted to determine the overall ideal adjustment goal of the forestry industry in state-owned forest areas. Therefore, multi-objective decision-making is performed to maximize the overall benefits of the forestry industry system in the forest region. To optimize the overall benefits of the forestry industry system in forest areas, three objectives are determined, ${f}_{1}(x)$, ${f}_{2}(x)$, and ${f}_{3}(x)$, which respectively represent the area of public welfare forest, multi-functional forest, and commercial forest. The linear weighting method is used for multi-objective decision-making, and its objective function reads:

9 $$ {\text{maxU}} = \gamma_{1} f_{1} \left( x \right) + \gamma_{2} f_{2} \left( x \right) + \gamma_{3} f_{3} \left( x \right) $$

10 $$ V_{n} = \left( {V - \alpha V_{c} } \right)\left( {1 + p} \right)^{n} - V_{c} \cdot \frac{{\left( {1 + p} \right)^{n} - \left( {1 + p} \right)}}{p} - \beta V_{c} $$

${V}_{n}$ represents the forest volume after *n* years; $V$ indicates the actual stand volume; $p$ means the stand productivity; ${V}_{c}$ stands for the annual quantitative cutting volume. U represents the total output value of forestry, ${\gamma }_{1},{\gamma }_{2},{\gamma }_{3}$ are the coefficients of different objective functions.

The area of 10 forest management type groups of the multi-functional forest, commercial forest, and public welfare forest in two stages are taken as decision variables, and the decision variables of artificial forest in the same two stages are constant. MOO constraints are set. The constraints in the nonlinear MOO process principally include: (1) constraints on the total area of forestry land; (2) Area constraints of each stand type; (3) growth constraints; (4) timber production constraints; (5) forest evolution state constraints.

### Data selection and processing

The data are from the panel data of the Comprehensive Statistical Data of Heilongjiang Forest Industry from 2015 to 2021. The research mainly selects input and output indicators for calculation. According to the principle of scientificity, comprehensiveness, representativeness, and practicability of index system construction, the comprehensiveness and importance of the forestry industry are taken into account. Combined with the index construction content in Ke et al.^30^, the total output value of forestry in Heilongjiang state-owned forest areas was taken as the output variable. The input variable selected the labor input variable and capital input variable, the former selected the number of employees at the end of the year, and the latter selected the cumulative investment completed since the beginning of the year. Considering that the area of forestry land is updated every five years, the land input variable is not included in the research measurement scope.

Considering the above factors, the total output value of the state-owned forest area of Heilongjiang is selected as the output variable, which can provide a comprehensive evaluation of the overall industrial development and economic contribution. Although the total forestry output value cannot directly reflect all aspects of industrial transformation, it is a relatively feasible and reasonable choice to select this value as the output variable based on considering the availability of data, the construction principle of the research index system, and the selection of other variables.

## Results

### Efficiency analysis of forestry industry transformation

**Figure 1:**
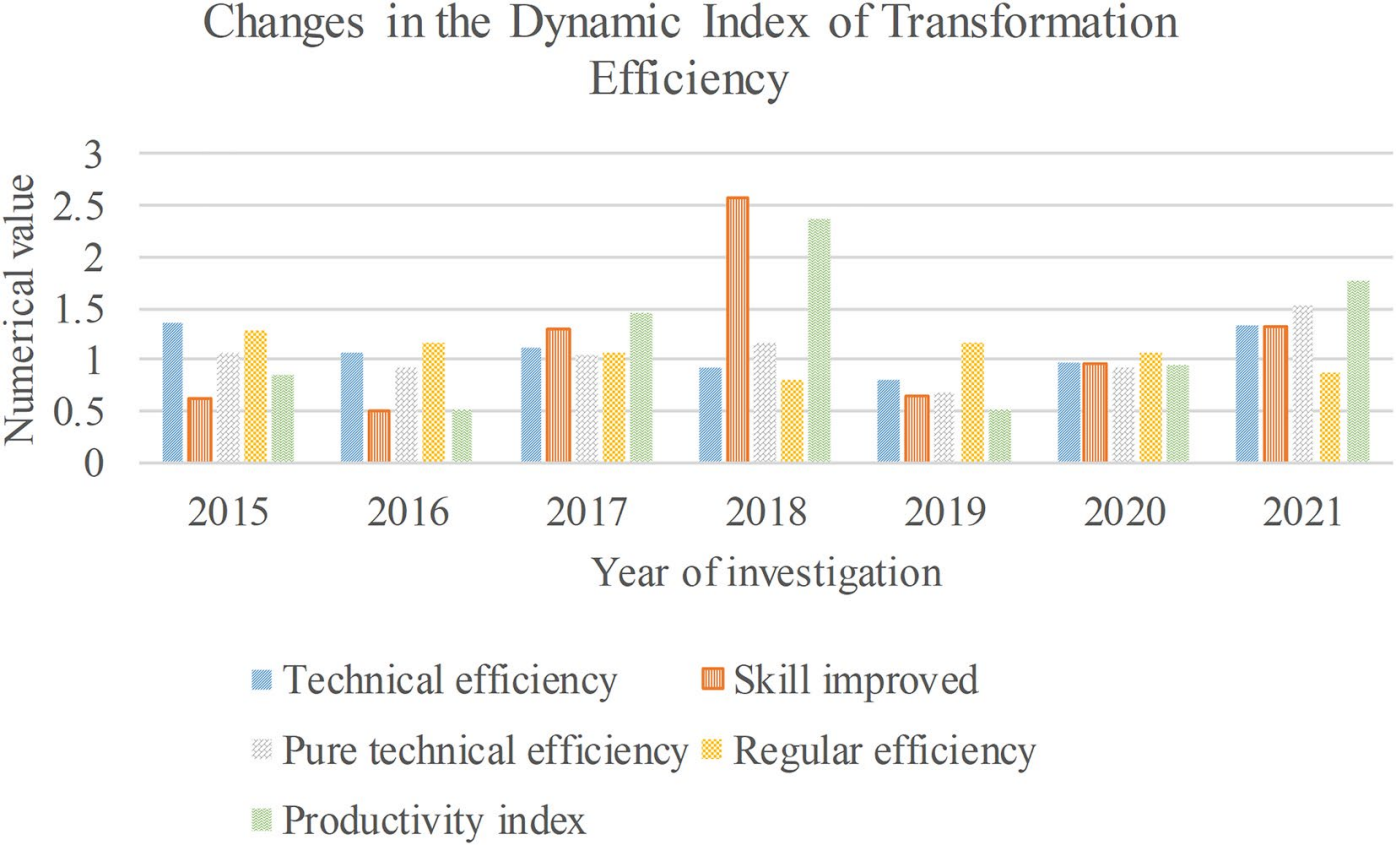
Dynamic index change of transformation efficiency.

This paper analyzes the dynamic index changes of forestry industry transformation efficiency in state-owned forest areas. The results are shown in Fig. 1:

Figure 1 denotes that during 2015–2018, the scale efficiency decreased yearly, but they were all greater than 1. The scale efficiency in 2019 was 0.801, less than 1. Until 2021, the scale efficiency showed an increasing state, and the efficiency was greater than 1, showing an overall optimistic state. The PTE decreased slightly in 2017, while in 2016 and 2018, the PTE was greater than 1. By the end of 2021, the efficiency value had increased to 1.538.

### Overall evaluation of forestry industry transformation efficiency in Heilongjiang state-owned forest areas

**Figure 2:**
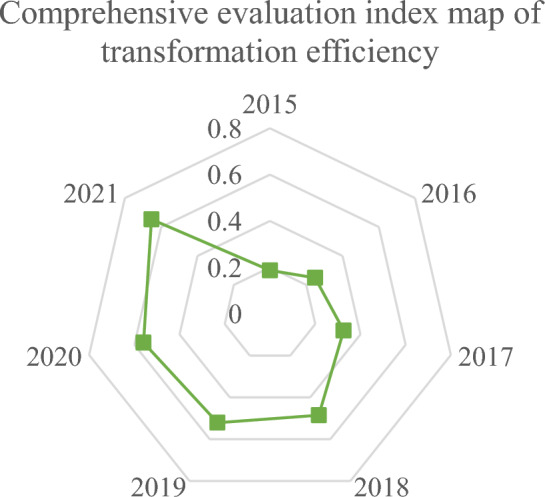
Survey results.

According to the forestry industry transformation’s evaluation system in Heilongjiang state-owned forest areas, the comprehensive evaluation index of forestry transformation efficiency in forest areas is calculated. Figure 2 presents the results:

The calculation results in Fig. 2 demonstrate that from 2015 to 2021, the transformation performance of the forestry industry in key state-owned forest areas in Heilongjiang has shown a good trend. The degree of volatility has gradually increased from a low of 0.1873 in 2015 to 0.6534 in 2021. The transformation of the forest area has experienced a development process of very poor—poor—qualified—medium. Factors such as policy adjustments, market demand, and resource availability can influence this phased change.

**Figure 3:**
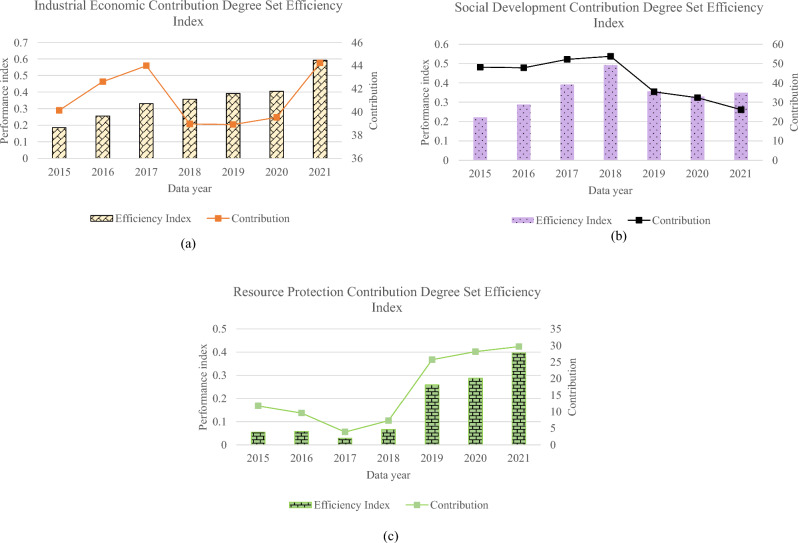
Evaluation result chart (**a**) industrial result chart; (**b**) social development result chart; (**c**) resource protection result chart.

The efficiency index of industrial development, social development, and resource protection from 2015 to 2021 and their contribution to the transformation of the forestry industry are further analyzed. The results are displayed in Fig. 3:

Figure 3 depicts the contribution trend of forestry industry transformation efficiency in Heilongjiang state-owned forest areas from 2015 to 2021, providing in-depth insight. The contribution of the industrial economic subsystem is stable, indicating that the industrial sector plays a key role in driving the development of industries such as forest product processing and wood production. In stark contrast, the contribution of the social development subsystem has weakened significantly, likely reflecting social challenges such as employment and farmers' incomes. Furthermore, the contribution of the resource conservation subsystem is in the form of “√”, and the initial successful investments and efforts tend to decline over time, which may require more sustained investments and policy adjustments to ensure sustainable resource management. Generally, the industrial economy subsystem is in the advanced position, the social development subsystem is in the middle position, and the resource protection subsystem is lagging behind. This highlights the key role of the industrial economy in driving transformational performance and the need to focus on the social development and resource conservation subsystems to achieve overall SD and improved performance.

### Analysis of nonlinear MOO results

**Figure 4:**
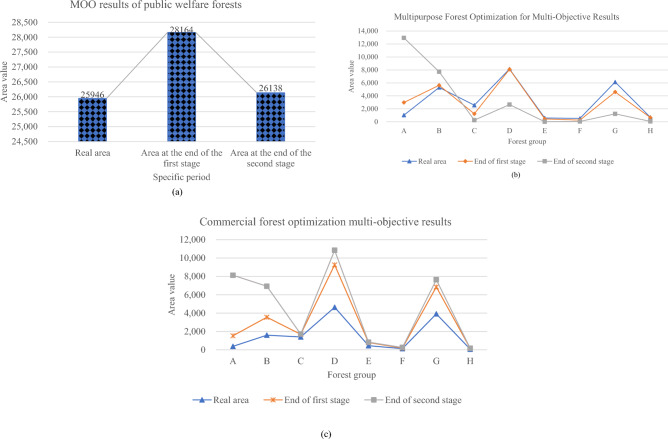
Result diagram of nonlinear MOO (**a**) result diagram of public welfare forest; (**b**) multi-functional forest result diagram; (**c**) commercial forest result diagram.

According to the given objective function and constraint equation, the forestry industry in the state-owned forest areas of Heilongjiang Province is solved by multi-objective programming. The obtained results are plotted in Fig. 4:

Figure 4 refers that the following suggestions can be proposed according to the calculation results. (1) For public welfare forests, the area in the two stages is higher than the actual area; (2) The area ratio of artificial forest to natural forest remains unchanged at about 3.5:6.5. Therefore, their area will not be adjusted in the two stages; (3) In the existing forest structure, the areas of public welfare forests, multi-functional forests, and commercial forests account for 31.40%, 40.3%, and 28.47%, respectively. At the end of the first stage, the percentage of public welfare, multi-functional, and commercial forests is 34.08%, 38.83%, and 27.9%, respectively. At the end of the second stage, public welfare forests, multi-functional forests, and commercial forests accounted for 31.63%, 40.8%, and 28.29%, respectively. In the two stages, the proportion of the public welfare forest area has increased compared with the initial area. Thus, the forestry industry in state-owned forest areas should be planned according to the suggestions that the proportion of public welfare forests, multi-functional forests, and commercial forests is 35.2%, 38.8%, and 26%, respectively.

### Discussion

This work aims to improve the effectiveness of the forestry industry transformation and promote the SD of social forest resources and the industrial economy. The result denoted that the transformation performance of the forestry industry in the Heilongjiang state-owned forest areas showed a positive development trend between 2015 and 2021. The average values of comprehensive production efficiency, technical efficiency, and scale efficiency are all less than 1, indicating that there is still potential to improve production efficiency. However, scale efficiency and PTE present an increasing trend, and PTE increases yearly, reaching a peak. The performance evaluation index of forestry transformation gradually increased from very poor to medium level, which showed a good situation of forestry industry transformation. The industrial economy subsystem contributes the most to the transformation performance. In contrast, the resource protection subsystem needs to catch up, which emphasizes the vital role of the industrial economy.

Besides, many studies have provided crucial references for this goal. First, Lu et al. (2021) discussed the impact of management risk perception and influencing factors on corporate social responsibility disclosure in China’s forestry sector, and improved the SD of forestry. On account of the survey data of 214 managers from Chinese forestry enterprises, the background of managers was analyzed through a two-stage model. It includes the relationship between six variables (gender, age, education level, degree and major, years of serving as managers, and work experience) and managers' risk perception of corporate social responsibility disclosure. The analysis of the two-stage model shows that the influencing factors of the two stages of risk perception are different^31^. Chen and Hassan ^32^ took China’s A-share listed companies from 2009 to 2018 as a sample. They used another method to examine the impact of carbon emissions trading on the financial performance of companies at the micro level. Evidence shows that although implementing carbon emissions trading has reduced the value of the current capital market, it has effectively improved the total asset-liability ratio of enterprises. Besides, in areas with a strict legal environment, the improvement effect of the total asset-liability ratio is more prominent. In areas with a loose legal environment, the reduction effect of capital market value is more significant. It also provides crucial support for the forestry industry transformation^32^.

The following are targeted suggestions to improve the transformation efficiency of Heilongjiang’s state-owned forest areas. First, the public welfare forest size should be increased in two phases to ensure that the proportion of the forests in the overall forest land utilization aligns with the planning recommendations. Second, the ratio of artificial forest to natural forest should be maintained at 3.5:6.5, with a slight adjustment. Third, according to the existing forest stand, the percentage of the area of public welfare, multi-functional, and commercial forests should be planned according to the suggestions, among which the proposed proportion of three forests is 35.2%, 38.8%, and 26%. Furthermore, technological innovation and personnel training should be strengthened, advanced production technology and management methods should be introduced, and labor productivity and resource utilization efficiency should be improved. The transformation efficiency of Heilongjiang state-owned forest areas is further promoted to achieve SD goals.

## Conclusion

This work analyzes the current transformation efficiency evaluation of the forestry industry in Heilongjiang state-owned forest areas to provide further suggestions for the transformation here. The internal nonlinear multi-objective problems of forestry in state-owned forest areas are optimized to help the development of the forestry industry. First, the forestry transformation efficiency evaluation method in forest areas is designed according to the DEA method. The evaluation index system of forestry industry transformation efficiency in Heilongjiang state-owned forest areas is further designed. Moreover, nonlinear MOO constraints are designed according to the current situation of the forestry industry in Heilongjiang state-owned forest areas. Relevant data are cited to evaluate the transformation efficiency of the forestry industry in Heilongjiang state-owned forest areas. The results reveal that the average comprehensive production, technical, and scale efficiencies of Heilongjiang state-owned forest areas from 2015 to 2021 are less than 1 and could be more effective. The overall industrial transformation of state-owned forest areas is optimistic. Technical efficiency increases gradually with the increase of years. At the end of 2021, the technical efficiency value peaked. The industrial economy subsystem has contributed more to the forestry industry transformation in state-owned forest areas. In the MOO of the forestry industry in state-owned forest areas, forest areas should be planned according to the proportion of public welfare forests, multi-functional forests, and commercial forests of 35.2%, 38.8%, and 26%, respectively. The research needs to be more field assessment, and relying on data analysis alone is likely to lead to experimental results errors. Further field visits will be conducted to supplement the experiment. Moreover, the research scope is small, and the representativeness of the research results is not strong. Therefore, future research will focus on expanding the research scope and improving the representativeness and contribution of the research results. This work can provide relevant suggestions and references for the follow-up forestry industry transformation in state-owned forest areas.

### Supplementary Information

Supplementary Tables.

## Data Availability

All data generated or analyzed during this study are included in this published article [and its supplementary information files]. If someone wants to request the data from this study please contact the Corresponding author. (Yude Geng, gengyude@nefu.edu.cn).
